# Ocular Surface Microbial Flora and Photorefractive Keratectomy

**DOI:** 10.1155/2022/5029064

**Published:** 2022-05-25

**Authors:** Alireza Peyman, Mehdi Bazukar, Tahmineh Narimani, Majid Mirmohammadkhani, Mohsen Pourazizi

**Affiliations:** ^1^Isfahan Eye Research Center, Department of Ophthalmology, Isfahan University of Medical Sciences, Isfahan, Iran; ^2^Department of Microbiology, School of Medicine, Isfahan University of Medical Sciences, Isfahan, Iran; ^3^Social Determinants of Health Research Center, Semnan University of Medical Sciences, Semnan, Iran; ^4^Department of Epidemiology and Biostatistics, School of Medicine, Semnan University of Medical Sciences, Semnan, Iran

## Abstract

**Purpose:**

To assess the influence of photorefractive keratectomy (PRK) on ocular surface microbial flora.

**Methods:**

A prospective study was conducted on patients who underwent PRK. The samples were taken from the inferior conjunctival fornix using a sterile swab, immediately before surgery, and then within three months following the PRK. The samples were tested using three culture mediums including blood agar, chocolate agar, and eosin methylene blue agar.

**Results:**

Thirty-five eyes of 35 patients including 19 females (54.3%) with a mean age of 24 ± 3.2 years were enrolled. The culture-positive rate was 15/35 eyes (42.9%) preoperative and 17/35 (48.6%) postoperative samples (*P*=0.47). The most common microorganisms isolated from preoperative samples were coagulase-negative *Staphylococcus* (CoNS) spp. in 14 (40%) samples, followed by *Streptococcus* spp. in 2 (5.7%), and *Staphylococcus aureus* in one (2.9%). Postoperative microorganisms isolated from conjunctival samples were CoNS spp. in 15 (42.9%), *Streptococcus* spp. in 3 (8.6%), and *Staphylococcus aureus* in one (2.9%), and *Corynebacterium* spp. in one (2.9%).

**Conclusion:**

This study indicated that there is not any remarkable difference in microorganisms isolated from conjunctival samples three months after PRK.

## 1. Introduction

Photorefractive keratectomy (PRK) is one of the popular refractive procedures using the excimer laser for the ablation of the anterior corneal tissue. Several complications can occur during and after PRK. Common short-term complications included pain, delayed visual recovery, and haze. Undercorrection or overcorrection, inability to tolerate contact lenses, light sensitivity, regression, decentration, haze, corneal ectasia, and dry eye are among the most common long-term complications [1–4].

Although with a low prevalence, the infection has remained one of the most potential complications of PRK in both short- and long-term periods [2, 5–8].

Conjunctival flora plays a defensive role as protects from colonization of pathogens. The pathogens responsible for the infection are mostly gram-positive ones [5, 9, 10], while the type of the microorganisms may be affected by age, gender, workplace, environment, underlying disease, and hospitalization [5, 9, 11].

Another aspect that seems to affect the conjunctival flora and leads to infections is the manipulation of the ocular surface through corneal refractive surgeries which can cause a reduction of functional meibomian glands, and therefore infection development [12, 13]. Contamination of contact lenses and probably the preoperative use of antibiotics are other factors that may be related to post-PRK infections [14–16].

To the best of our knowledge, the studies conducted previously to assess the factors related to the post-PRK infections have been almost conducted during the periods that the patients were under antibiotics, steroids, or other treatment, while in the current study, we have aimed to find the effects of PRK on the conjunctival bacterial flora within three months following the procedure.

## 2. Material and Methods

### 2.1. Participation and Study Design

In this prospective before and after study, subjects who were candidates for the PRK and referred to the Feiz Hospital, the ophthalmology center affiliated with the Isfahan University of Medical Sciences, were included. The Ethics Committee of Isfahan University of Medical Sciences primarily confirmed the protocol of the study (IR.MUI.REC.1396.3.042).

The participants were patients with refractive errors aged above 18 years who were candidates for PRK. The study protocol was entirely explained to the included participants, and they were requested to present the signed consent form of participation in the study.

History of underlying chronic diseases (e.g., diabetes mellitus and hypertension), chronic use of systemic immunosuppressive agents, oral antibiotics use within three weeks before the surgery, smoking and/or alcohol use, chronic use of topical or oral corticosteroids, and pregnancy were considered as the unmet criteria. Those who required reoperation or presented conjunctival infection within three months following the surgical procedure were excluded from the study.

Sequential inertial sampling was used to enroll the study population. One eye of each participant was selected randomly by using a random number generator statistical table.

### 2.2. Surgical Procedures

All surgeries were performed by a single skilled surgeon (A.P.). After instilling topical anesthesia (tetracaine 0.5%, SinaDarou, Iran), an eyelid speculum was inserted. The surface corneal epithelium in a 9 mm diameter area was loosened using a 20% alcohol solution and removed using a blunt spatula (Hockey knife). Surgery was performed using a Technolas 217z100 excimer laser system (Bausch & Lomb). PRK protocols were described elsewhere in detail [1, 4].

### 2.3. Conjunctival Cultures

Conjunctival sampling was performed for one eye of each patient before surgery and 3 months after. To assess the flora, in the operation room and immediately before anesthesia by tetracaine 0.5%, a sterile wet swab was administered to obtain the samples from the inferior conjunctival fornix of one eye. The swabs had no contact with the lids and were immediately added to three culture mediums including blood agar, chocolate agar, and eosin methylene blue agar. The mediums were prepared at most within twelve hours before the use. All of the plates were placed in the incubator for an hour at 37 centigrade degrees and then checked by a microbiologist daily for three days. Standard microbiologic studies, including gram staining, colony morphology study, motility assessment, oxidase, and catalase tests were performed. Besides, to make definite microorganism identification, automated identification kits were utilized. Within three months following the surgery, the patients were referred for the follow-up and conjunctival sampling was performed with similar techniques and assessments.

### 2.4. Statistical Analyses

Descriptive data were presented in mean, standard deviation, percentages, and absolute numbers. McNemar's test was used to compare the pre- and postoperative data. The data were analyzed in SPSS (version 22) software (Statistical Procedures for Social Sciences, Chicago, Illinois, USA). *P* values less than 0.05 were considered significant in all the tests.

## 3. Results

Thirty-five eyes of 35 patients (19 females and 16 males; mean age, 24 ± 3.2 years) were enrolled. The conjunctival culture grew organisms in 15 of 35 samples (42.9%) preoperative. Of the 15 samples with positive culture results, 2 (13.3%) had mixed cultures with two strains isolated (each for two microorganisms). After 3 months of operation, the conjunctival culture grew organisms in 17 of 35 samples (48.6%). Of the 17 samples with positive culture results, 3 (17.6%) had mixed cultures with two strains isolated (each for two microorganisms). There was no significant difference between pre- and postoperative culture-positive rates (*P*=0.47).

The most common microorganisms isolated from preoperative samples were coagulase-negative *Staphylococcus* (CoNS) spp. in 14 (40%) samples, followed by *Streptococcus* spp. in 2 (5.7%) and *Staphylococcus aureus* in one (2.9%) (Table 1). Postoperative microorganisms isolated from conjunctival samples were CoNS spp. in 15 (42.9%), *Streptococcus* spp. in 3 (8.6%), and *Staphylococcus aureus* in one (2.9%), and *Corynebacterium* spp. in one (2.9%) (Table 1).

A comparison of preoperative versus postoperative positivity of the cultures showed no statistical differences in the numbers of microorganisms (*P* > 0.05). Table 1 presents microorganisms isolated from conjunctival flora before and after PRK.

## 4. Discussion

The current study was conducted to determine the influence of PRK, as one of the most popular refractive surgeries, on the normal flora of the conjunctiva. We found that the conjunctival flora was not remarkably affected by this procedure after 3 months. Besides, CoNS was the most common type of microorganism found both preoperatively and postoperatively.

In our study, positive bacterial growth was detected in about 43% of all the samples before surgery which was in the range presented by other studies. Tao et al. presented the range of 19–83% of positive isolations based on the age categories [12]. The other study by Sthapit et al. assessed 200 patients and presented a positivity of 78.5% [5]. This diversity can be attributed to the type of patient selection in different studies, as we have excluded patients under treatment with any agent that can probably influence the flora including antibiotics, immunosuppressive agents, and steroids. Other factors include the past medical history of diabetes, use of contact lenses, and eyelid deformities. Genetic factors, occupation settings, and diversity in the geographical distribution of the microorganisms are factors that should not be underestimated [13, 17]. In general, almost half of the healthy population has positive cultures of the conjunctiva; this is the point that all of the studies are in stock.

Preoperative assessment of microorganisms in our study revealed CoNS as the most prevalent culture followed by *Staphylococcus aureus*. These findings were in agreement with the study of Ansari et al. that assessed 4391 cultures and presented gram-positive organisms in 94.2% of the cultures while the remaining 5.3% were gram-negative. CoNS was positive in 88.3%, followed by *Streptococcus* species in 23.1%, and *Staphylococcus aureus* in 10.2% of the cultures [18]. Capriotti et al. conducted another study and showed aerobic gram-positive bacteria in 62%, CoNS in 28.6%, and *Staphylococcus aureus* in 19.9% in a total number of 276 cases [9]. Singer et al. reported *Staphylococcus epidermidis* in 40% and *Staphylococcus aureus* in 30% of the assessed cases. These microorganisms are the familiar residents of skin and mucous membrane flora and are acquired in conjunctiva from the adjacent eyelid or hands [11].

Yuksel et al. evaluated microbiologic cultures of bandage contact lenses (BCL) used following corneal collagen cross-linking and PRK in Turkey. They presented that 12.2% of the lenses were positive postoperatively [14]. The other similar study by Dantas et al. performed a similar study and analyzed the microbiological study of the contact lenses within three days following the operation. They declared 8.6% of positivity among the studied population [19]. Liu et al. evaluated the bacterial contamination of BCL after PRK, and reported 6.67% of positivity among cultured isolations [20]. Mentioned studies were entirely performed within a few days following the procedure while ours was performed within three months. We found postoperative positive cultures in about 49% of the cases, which was slightly more than the preoperative assessment but insignificant. Besides, the rate of 49% is remarkably higher than other studies in this regard, which may be attributed to the time of assessment, as preoperative medications may affect the conjunctival colonization, or to the number of studies' populations. Despite mentioned reasoning, we think that PRK does not affect the conjunctival flora.

Jung et al. raised a theory about the probability of infection incidence following the PRK surgery because of ocular surface manipulation, and reduction of meibomian glands function following the surgery [21]. This theory was not favored by other studies, as Dantas et al. presented similar outcomes to our preoperative assessments, and only a patient out of 81 ones was positive for gram-negative bacillus [19]. Similar presentations were reported by Barry et al. [22], and Yuksel et al. [14] as well. However, in contrast to our study, mentioned reports were performed within a few days following the surgical procedures.

To the best of our knowledge, the current study is the first to assess PRK effects on the conjunctival flora within a considerable interval of three months. There are several limitations in the current study that should be noted. First, we did not evaluate other microorganisms such as fungi. Second, an antibiogram was not performed in our study. Other limitations are that we evaluated the conjunctival flora before and 3 months after PRK. It does not clear whether the changes are the permanent effect of PRK or alter with time. Further studies should be conducted to evaluate the changes of conjunctival flora during each month after PRK by serial culture.

In conclusion, gram-positive bacteria were the most common isolated microorganism derived from the conjunctival surface, whether preoperatively or postoperatively. In this study, no remarkable difference in microorganisms isolated from conjunctival samples was found three months after PRK, so it seems PRK had no significant effect on conjunctival flora at least in the first 3 months of follow-up. Further clinical research with more follow-up period and larger sample size is needed to clarify the effects of PRK on conjunctival flora and the potential risk of infection postprocedure.

## Figures and Tables

**Table 1:** Microorganisms isolated from the ocular surface before and after PRK.

Microorganisms	Samples, *n* (percentage)				*P* value
	Preoperation		Postoperation		
CoNS	14	(40.0)	15	(42.9)	0.81
SA	1	(2.9)	1	(2.9)	1.00
Strep	2	(5.7)	3	(8.6)	0.64
Coryn	0	(0.0)	1	(2.9)	—

CoNS: coagulase-negative *Staphylococcus*; SA: *Staphylococcus aureus*; Strep: *Streptococcus*; Coryn: *Corynebacterium* spp.

## Data Availability

The data are available on request.
